# Vascularised endosteal bone tissue in armoured sauropod dinosaurs

**DOI:** 10.1038/srep24858

**Published:** 2016-04-26

**Authors:** Anusuya Chinsamy, Ignacio Cerda, Jaime Powell

**Affiliations:** 1 University of Cape Town, Department of Biological Sciences, Private Bag X3, Rhodes Gift, 7700 South Africa; 2 CONICET, Instituto de Investigación en Paleobiología y Geología, Universidad Nacional de Río Negro, Museo Carlos Ameghino, Belgrano 1700, Paraje Pichi Ruca (predio Marabunta) 8300, Cipolletti, Río Negro, Argentina; 3 CONICET, Facultad de Ciencias Naturales Universidad Nacional de Tucumán, Miguel Lillo 205, (4000) Tucumán, Argentina

## Abstract

The presence of well-vascularised, endosteal bone in the medullary region of long bones of nonavian dinosaurs has been invoked as being homologous to medullary bone, a specialised bone tissue formed during ovulation in birds. However, similar bone tissues can result as a pathological response in modern birds and in nonavian dinosaurs, and has also been reported in an immature nonavian dinosaur. Here we report on the occurrence of well-vascularised endosteally formed bone tissue in three skeletal elements of armoured titanosaur sauropods from the Upper Cretaceous of Argentina: i) within the medullary cavity of a metatarsal, ii) inside a pneumatic cavity of a posterior caudal vertebra, iii) in intra-trabecular spaces in an osteoderm. We show that considering the criteria of location, origin (or development), and histology, these endosteally derived tissues in the saltasaurine titanosaurs could be described as either medullary bone or pathological bone. Furthermore, we show that similar endosteally formed well-vascularised bone tissue is fairly widely distributed among nondinosaurian Archosauriformes, and are not restricted to long bones, but can occur in the axial, and dermal skeleton. We propose that independent evidence is required to verify whether vascularised endosteal bone tissues in extinct archosaurs are pathological or reproductive in nature.

Typically endosteally formed bone in vertebrates tends to be avascular lamellar or parallel-fibered bone tissue^1^,^2^. However, endosteally formed tissue called medullary bone, is known to occur in the medullary cavities of birds^3^,^4^,^5^,^6^,^7^,^8^,^9^,^10^, and has been reported in nonavian dinosaurs^9^,^11^,^12^. However a similar endosteally formed tissue is also known to be pathological in extant birds^13^,^14^. Thus, the occurrence of well-vascularised endosteally formed bone tissue in nonavian archosaurs has been interpreted as both medullary bone^9^,^11^,^12^, as well as pathological bone^15^,^16^,^17^, ^19^,^20^,^21^,^22^,^23^ (Table 1). Most of these reports are from long bones of nonavian dinosaurs, although, Reid^15^ described a well-vascularised endosteally formed bone as a pathology (possibly caused by cancerous metastasis) in an internal cavity of a Wealden sauropod vertebra. Furthermore, it appears that such bone tissue occurs quite widely among non-dinosaurian archosaurs^17^,^18^,^19^, and they have been likewise referred to as pathological, reproductive or of unknown origin^18^ (Table 1). Researchers have attempted to use various criteria to distinguish medullary bone^9^,^11^,^12^ from pathological bone^16^ or other endosteally derived medullary bone-like tissues^18^. The problem nevertheless still persists, and appears to be fertile ground for fostering research in this area^24^,^25^.

In the current study we describe the occurrence of well-vascularised endosteal bone tissue in an osteoderm, a vertebra and a metatarsal of armoured titanosaur sauropods from the Upper Cretaceous of Argentina. Here we propose the likely causes of its formation and the implications for the identification of endosteal tissues homologous to avian medullary bone in fossil archosaurs.

## Materials and Methods

The bone histology of a metatarsal (PVL 4017–127), a distal caudal vertebra (PVL 4017–140) and an osteoderm (PVL 4017−113) of saltasaurine titanosaurs (possibly *Saltasaurus loricatus*, see supplementary information) are described here. This material forms a subset of a larger sample comprising of seventy-four bones representing various parts of the skeleton of titanosaur sauropods (Sup Table S1), including both axial and appendicular elements. The three specimens described here were collected from the Upper Cretaceous (? late Campanian–Maastrichtian) sediments of the Lecho Formation at the locality of El Brete (south of Salta Province, Argentina)^26^,^27^, and were assigned to *Saltasaurus loricatus* on the basis of morphology and/or close association with diagnostic elements^26^,^27^,^28^. Nevertheless, since autapomorphic features are absent from these elements, we only refer to them as indeterminate saltasaurine titanosaurs. It is worth noting though that even if our diagnosis that they are *Saltasaurus loricatus* is incorrect, the elements are all from Saltasaurinae, and do not detract from our findings. The preparation of the histological sections was carried out in the Departamento de Geología de la Universidad Nacional de San Luis in Argentina. Thin sections were prepared using the method outlined by Chinsamy and Raath^29^ (see sup Fig. S1 for location of sections) and they were studied using a petrographic polarizing microscope (Nikon E200 pol). Nomenclature and definitions of structures used in this study are derived from Francillon-Viellot *et al.*^1^ and Chinsamy-Turan^2^.

## Results

**Figure 1:**
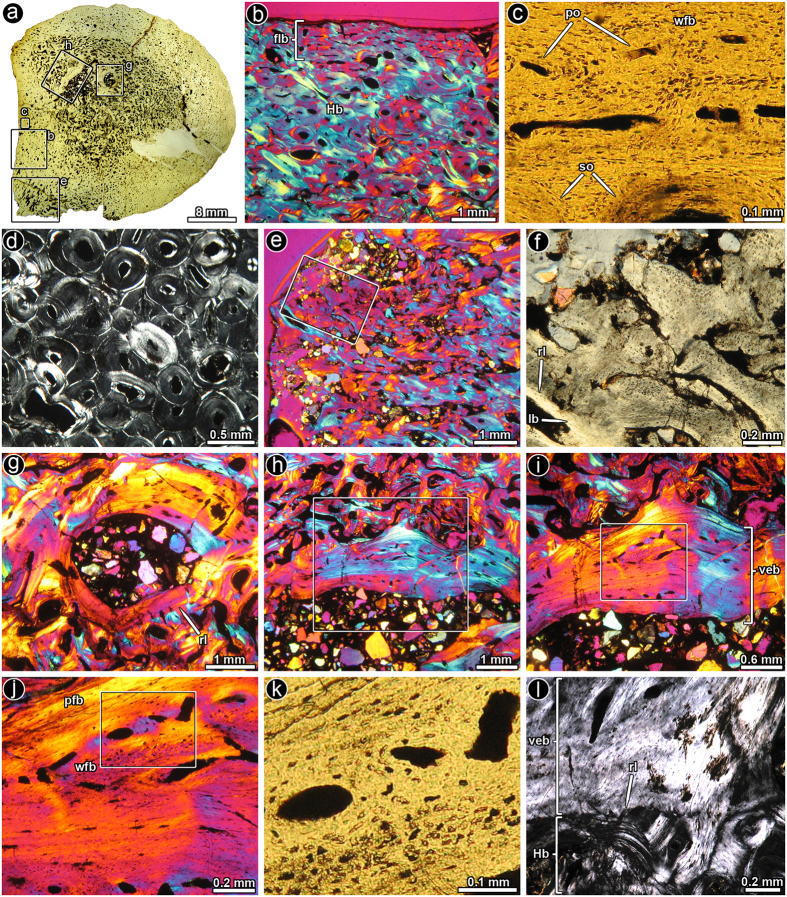
Bone histology of saltasaurine titanosaur metatarsal PVL 4017–127. (**a**) Complete transverse section of the metatarsal. Note the rather compacted general appearance of the bone, even in the cancellous region. The external cortex exhibits a shallow excavation in portion of the sample (left bottom margin), which also exhibits a high degree of porosity. (**b**) General view of the cortical bone, which is mostly composed of dense Haversian bone tissue. A thin layer of unremodelled primary fibro-lamellar bone is observed at the subperiosteal region. (**c**) Detail of primary fibro-lamellar bone tissue at the outer cortex. (**d**) Enlarged view of the dense Haversian bone tissue formed at the mid-cortex. (**e**) General view of the external cortex showing a highly porous tissue. (**f**) Detail of the same region showing a highly fibrous primary bone tissue and some secondary osteons. (**g**) Internal cavity in the medullary region, which is coated a thick layer of parallel and lamellar bone tissue. (**h**) General view of the medullary region in which a thick band of vascularised endosteal bone is formed around a large internal cavity. (**i**) Detail of the same region in h. Note the roughly stratified pattern of the vascularised endosteal bone tissue, with successive layers of woven- and parallel-fibered bone tissue. (**j**) Detail of the same region showing the transition between a poorly vascularised parallel-fibered bone and more vascularised woven-fibered bone in the vascularised endosteal bone. (**k**) Detailed view of the woven-fibered matrix in the vascularised endosteal bone tissue. Note the presence of well-developed primary osteons. (**l**) Detail view of the vascularised endosteal bone tissue. The resorption line between the dense Haversian bone and the vascularised endosteal bone demonstrate the secondary nature of the latter tissue. Images (**a,c,k**): normal transmitted light; (**d,l**): cross polarized light; (**f**) single plane polarized light; (**b,e,g-j**): cross polarized light with lambda compensator. Abbreviations: lb, lamellar bone; flb, fibro-lamellar bone tissue; Hb, Haversian bone; pfb, parallel-fibered bone tissue; po, primary osteons; rl, resorption line; so, secondary osteons; veb, vascularised endosteal bone; wfb, woven-fibered bone tissue.

Except for the metatarsal, caudal vertebra and osteoderm, the histology of 74 other postcranial bones of sauropod titanosaurs studied (see supplementary Table S1) did not exhibit any unusual features (i.e., vascularised endosteal bone). Thus, here we limit our report to the atypical features observed in the three bones mentioned. In the sampled metatarsal (Fig. 1a), the transverse sections obtained from the mid-shaft region shows a thin outer band of primary bone tissue (Fig. 1b,c), while the rest of compacta is extremely highly remodelled and reaches dense Haversian proportions (Fig. 1b,d). The innermost parts of the cortex has a large amount of trabeculae which tend to have thick deposits of lamellar bone and the interstitial tissue also predominantly consists of lamellar bone, although there is also some compacted coarse cancellous bone present. The medullary region exhibits a rather compacted appearance under low magnifications. In a distinct portion of the section, the cancellous bone tissue extends from the medullary cavity to the outer cortex (Fig. 1a). At level of the outer cortex of this region, a rather unusual excavation is evident and the primary bone in this region is composed of coarse bundles of mineralized fibres (Fig. 1e,f). A high degree of porosity is also evident in this area. Some distinct larger cavities are observed at the medullary region (Fig. 1g,h). These cavities show a distinctive resorptive margin, which is followed by a secondary deposition (sensu Francillon-Vieillot^1^) of a tissue intermediate between parallel-fibered and woven bone tissue with some vascular spaces entrapped within the bone matrix (Fig. 1i,j). Some of these channels have narrow bands of lamellar bone deposits. Some regions around the large cavities possess thick bands of lamellar bone tissue, which alternates with a well-vascularised region of parallel and woven-fibered bone (Fig. 1j,k). Whereas osteocyte lacunae in the parallel-fibered matrix are flattened and oriented in parallel each other, those located in the woven-fibered bone possess irregular shapes and lacks spatial organization (Fig. 1k,l). In parts of the periosteal region, there is a large amount of resorption of the original dense Haversian bone with the subsequent deposition of lamellar bone. However, the lamellar bone has different orientations, and to some extent resembles compacted coarse cancellous bone.

**Figure 2:**
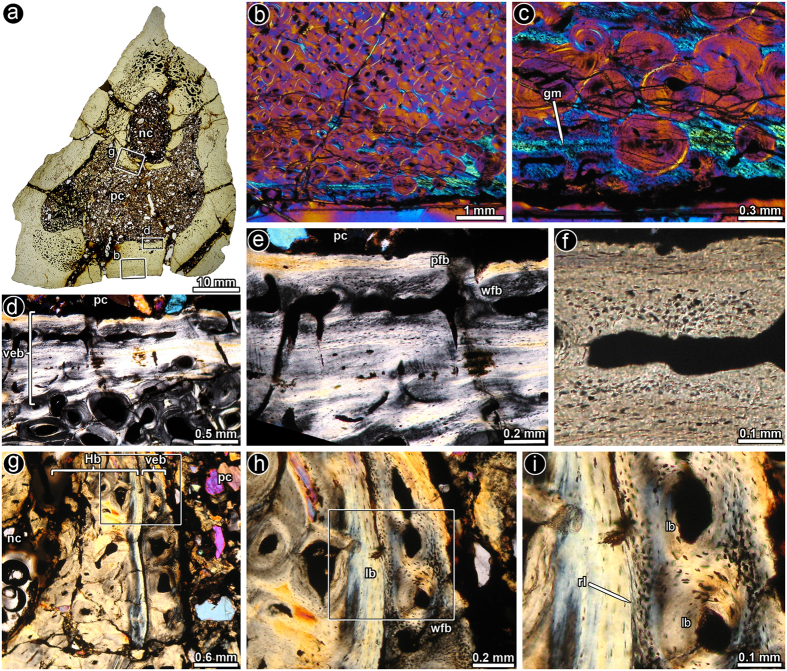
Bone histology of saltasaurine titanosaur distal caudal vertebra PVL 4017–140. (**a**) Complete transverse section of the vertebra. A large pneumatic cavity is formed in the centrum and part of the neural arch. (**b**) General view of the cortical bone, which is mostly composed of dense Haversian bone tissue. A thin layer of unremodelled primary bone is observed at the outer region. (**c**) Detail of primary bone tissue at the outer cortex. (**d**) General view of the inner cortex showing the bone tissue around the pneumatic cavity. Note the presence of a thick band of vascularised endosteal bone around a large internal cavity. (**e**) Detail of d. Note the stratified pattern of the vascularised endosteal bone tissue, with successive layers of woven- and parallel-fibered bone tissue. (**f**) Detail of the same picture showing the transition between a poorly vascularised parallel-fibered bone and more vascularised woven-fibered bone in the vascularised endosteal bone. Note the distinct differences in the osteocyte lacunae shape, density and arrangement. (**g**) Vascularised endosteal bone tissue and Haversian bone tissue below the neural canal. (**h**) Detail of g showing the transition between dense Haversian bone and vascularised endosteal bone. (**i**) Detail of h. The resorption line between the dense Haversian bone and the vascularised endosteal bone demonstrate the secondary nature of the latter tissue. Images (**a**,**f**): normal transmitted light; (**b**,**c**), polarized light with lambda compensator; (**d,e,g-i**): cross polarized light. Abbreviations: lb, lamellar bone; gm, growth mark; Hb, Haversian bone; nc: neural canal; pc, pneumatic cavity; pfb, parallel-fibered bone tissue; rl, resorption line; veb, vascularised endosteal bone; wfb, woven-fibered bone tissue.

In the caudal vertebra, three sections were prepared (two from the middle region and one from the posterior area). All three sections of the vertebra show a thick layer of endosteal bone that lines a large pneumatic cavity (Fig. 2a). The compact bone is almost entirely composed of dense Haversian bone tissue (Fig. 2b). Remains of periosteal bone, which grades from parallel-fibered (e.g. ventral cortex) to fibrolamellar bone (e.g. lateroventral region), are observed in outermost portion of the element (Fig. 2c). An atypical endosteal tissue is observed coating the large pneumatic cavity of the vertebra. The fibrillar organization of this unusual endosteal tissue appears to be variable and ranges from lamellar to parallel-fibered to woven bone (Fig. 2d–i). Cementing lines separate the well-vascularised endosteal bone from the “normal” lamellar bone tissue (Fig. 2g–i). Osteocyte lacunae are densely grouped in some areas and they exhibit an irregular shape and a chaotic organization i.e. quite different from the pattern observed in the surrounding lamellar bone tissue (Fig. 2f,i). The tissue often includes “primary” osteons, i.e. vascular channels surrounded by centripetally deposited lamellar bone tissue, as well as some channels showing obvious resorptive surfaces.

**Figure 3:**
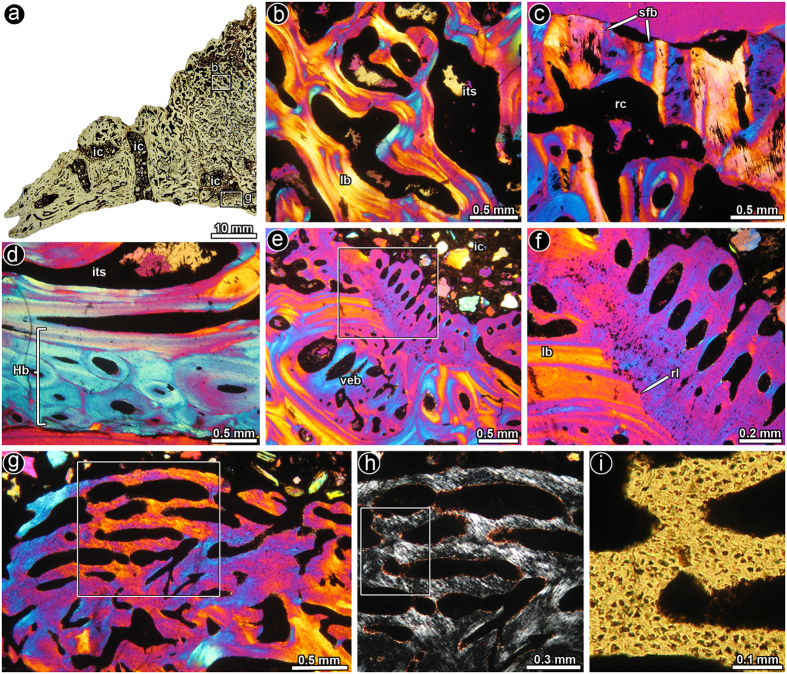
Bone histology of saltasaurine titanosaur osteoderm PVL 4017–113. (**a**) Half portion of the osteoderm in transverse section. The internal microanatomy of the elements is characterized by abundant cancellous bone and several large internal cavities. (**b**) Detailed view of the secondary cancellous bone tissue, which is entirely formed by secondary lamellar bone tissue deposited during different episodes of remodeling. (**c**) Remains of primary bone tissue at the outer cortex, which is formed by interwoven bundles of mineralized structural fibres (**d**) Enlarged view of the dense Haversian bone tissue formed at the internal cortex of the osteoderm. (**e**) General view of the inner cortex showing the bone tissue around the internal cavity. Note the presence of a thick band of vascularised endosteal bone is formed around a large internal cavity. (**e**) Highly vascularised endosteal bone is formed around a large internal cavity. Note that an adjacent resorption cavity has been also filled with vascularised endosteal bone. (**i**) Detail of e showing the abrupt transition between endosteal lamellar bone tissue and vascularised endosteal tissue. A distinctive resorption line demarcates this transition. (**g**) Highly vascularised endosteal bone is formed around a large internal cavity. (**h**) Detail of g showing the arrangement of intrinsic fibres. (**i**) Detailed view of the same picture showing the shape, density and arrangement of osteocyte lacunae in the vascularised endosteal bone. Images (**a**,**i**): normal transmitted light; (**b**–**l**): cross polarized light with lambda compensator; (**h**) polarized light. Abbreviations: lb, lamellar bone; Hb, Haversian bone; rc, resorption cavity; rl, resorption line; ic, internal cavity; its, intertrabecular spaces; veb, vascularised endosteal bone.

The sampled osteoderm is a conical plate for which a histological description was published by Cerda and Powell^28^. The thin sections obtained from the osteoderm reveal that the overall structure is rather cancellous (as opposed to being compact), with abundant inter-trabecular spaces lined by lamellar bone (Fig. 3a,b). Remains of primary bone tissue are preserved in some areas and consist of coarsely bundled mineralized structural fibers (Fig. 3c), which have been commonly observed in osteoderms of titanosaurs and ankylosaurs and originate from metaplasic ossification^23^,^28^,^30^,^31^. Dense Haversian bone is formed at the internal and lateral cortices (Fig. 3d). Large internal cavities are located near the deep cortex (Fig. 3e–g). Some of these cavities are lined with a highly vascularised secondary bone tissue (Fig. 3f–h). This unusual endosteal bone is distinct from the secondary lamellar bone formed in the cancellous bone and consists of parallel-fibered and woven bone tissue. The secondary nature of this endosteal bone is clearly revealed by the resorptive surface of the “normal” secondary lamellar bone from which the vascularised endosteal tissue is deposited (Fig. 3f). Osteocyte lacunae are densely grouped together and they exhibit globular to rounded shapes (Fig. 3i). Several inter-trabecular spaces located near the large cavities are also infilled with this unusual secondary bone tissue (Fig. 3e).

## Discussion

In the current study we were able to identify at least three ways in which the endosteal bone formed: i) The typical or most common type of endosteal bone tissue observed among many vertebrates and the saltasaurine specimens studied here is an avascular lamellar lining bone tissue^1^,^2^ (Supplementary Fig. S2–S6). ii) The second type of endosteal bone appears to be an extensively developed bone tissue consisting of vascularised lamellar/parallel-fibered and woven-fibered bone, and can sometimes alternate to form a stratified pattern (Fig. 2e). This tissue was observed mainly in the pneumatic cavities of the saltasaurine caudal vertebra and locally in the medullary cavity of the metatarsal. iii) The third type of endosteal bone tissue consisted of a highly vascularised parallel-fibered and coarsely fibered woven bone tissue. This is predominantly seen in the cancellous spaces and large channels in the osteoderm (Fig. 3e,g). In previous histological studies on dermal, axial and appendicular bones of titanosaur sauropods^28^,^32^,^33^,^34^,^35^,^36^,^37^,^38^,^39^,^40^,^41^,^42^ only the first type of endosteal bone tissues were reported, and no mention was made of the other endosteal tissue types. Furthermore, in our sample of 74 bones of titanosaurs we only found the second and third types of endosteally formed tissue in three bones. In addition, they have also not been reported in previous studies of more than 200 bones of other titanosaur sauropods studied histologically. These findings suggest that these endosteal tissue types are not common in the skeleton of titanosaurs, and they appear to be unrelated to cortical drift or biomechanical adaptive remodelling. However, it is interesting that similar vascularised endosteal tissues are known to occur among extant vertebrates under particular circumstances, i.e. in reproductively active avian females^4^,^8^ and under some pathological conditions^13^,^14^.

Among extant archosaurs, reproduction inflicts particular histological changes in the bone microstructure of females. In crocodiles, long bones and osteoderms of females (as opposed to males) are extensively reconstructed during egg laying due to resorption of calcium for the calcification of eggshells^43^,^44^,^45^,^46^. Breeding females of *Crocodylus niloticus*^46^, *Alligator mississipiensis*^47^ and *Crocodylus johnstoni*^48^ are reported to show extensive remodelling of their osteoderms, which often result in the obliteration of skeletal growth marks. On the other hand, among several extant bird taxa, prior to egg production females deposit a specially formed bone tissue (called medullary bone) within the medullary cavity of various bones in the skeleton, which acts as a reservoir for calcium during egg shelling^4^,^8^,^9^.

Four nonavian dinosaur taxa are reported to have a reproductive bone tissue homologous to that of avian medullary bone (Table 1). The identification of these tissues as medullary bone in these nonavian dinosaurs has been on the basis of location (within medullary cavities of long bones), origin (i.e. endosteally developed), and histology (vascularised woven bone tissue with its characteristic birefringence). Considering these criteria, it appears that the endosteally formed vascularised bone tissue formed in the saltasaurines described here could be medullary bone. However, these same criteria (location, origin and histology) also hold true for pathological bone. One of the diagnostic characteristics generally used to identify medullary bone in fossil taxa is its location within the medullary cavities of long bones. However, although medullary bone is predominantly located in long bones, it can also occur in various bones of the skeleton, including axial, and distal appendicular bones, as well as in pneumatic bones^49^. This means that the location of well-vascularised endosteal bone tissue within the vertebra and metatarsal of our study material could easily also fit the criteria for medullary bone. Our observation of this unusual bone tissue within the pneumatic cavity of the caudal vertebra, does not preclude it from being medullary bone since such bone has been located within pneumatic bones of birds^49^. However, pathological bone can also be found in any part of the skeleton and within medullary cavities. Thus, on the basis of location, our findings of highly vascularised endosteal bone tissue in the saltasaurine titanosaur bones could be interpreted as a pathology as well^16^. Considering the origin criteria, medullary bone develops from the endosteum of medullary cavities and other internal spaces and is under hormonal control in reproductive females^50^. It is not associated with any specialized periosteal bone tissue. Vascularised endosteally formed tissue is also produced as a result of a pathology e.g. avian osteopetrosis^10^,^16^,^22^ and during fracture healing processes^20^. Avian osteopetrosis often results in both a periosteal and an endosteal reaction, although there are reports of only endosteal reactive bone tissue being formed^13^. In the case of our samples, none of the bones show any signs of an external callus, and in addition, although the metatarsal shows some unusual histology in a localized periosteal part of the cortex, a distinctive periosteal reactive bone tissue is not observed. Other diseases, e.g., Paget’s disease, osteomyelitis, lytic metastatic lesions from cancers can also cause osteoblastic activity that can result in pathologically formed endosteal bone tissues^51^,^52^,^53^,^54^. Given that both medullary bone and various pathologies can result in well-vascularised endosteal bone tissue we cannot conclusively identify whether the tissues in the saltasaurine titanosaur vertebra and the osteoderm are homologous to medullary bone or whether they are pathological. The unusual histology of the metatarsal is also endosteally formed but in this case, it could be diagnosed as a pathological response (see below). Finally, taking into account the third criterion (histology), medullary bone has been characterized as a well-vascularised endosteally formed woven bone tissue^2^,^9^. Pathological bone on the other hand, can manifest as different kinds of bone tissue: for example, several diseases can result in well-vascularised woven bone tissue (e.g. metastatic cancer and osteomyelitis), and in diseases such as avian osteopetrosis the bone tissue is characterized by a high density of osteocyte lacunae, whilst Paget’s diseases is distinctive in having mainly remodelled lamellar kind of bone tissue^48^. In our saltasaurine titanosaur samples, the histological nature of the tissue rules out avian osteopetrosis and Paget’s disease. In addition, the lack of any fracture callus excludes fracture healing as the cause of the deposition. However, other diseases such as, osteomyelitis and metastatic cancer could have resulted in such anomalous endosteally formed vascularised bone tissue^55^.

Besides location, origin and histology, attainment of sexual maturity^11^,^12^,^18^ has also been used as an additional criterion for identification of medullary bone (since it is only formed in reproductively active females). The periosteal bone tissue of the saltasaurine titanosaur metatarsal and the caudal vertebra suggests that they were from still rapidly growing individuals which had not experienced a “slow down” in growth generally attributable to the attainment of sexual maturity.

Another interesting feature of the saltasaurine endosteally formed bone tissue is that unlike medullary bone, besides the well-vascularised endosteal woven bone, it also has a large amount of endosteally formed parallel-fibered and lamellar bone tissues which sometimes appear to be formed in cycles (Figs 1I and 2e). In some instances, such as in the vertebrae and the metatarsal, this tissue can result in a thick layer (Figs 1g and 3g) and leads to some compaction of the element. Therefore, “cyclically” formed endosteal bone in the vertebra and in the metatarsal could also be seen as being different to medullary bone usually described in the literature^2^,^9^.

Besides the presence of an abnormal endosteal bone tissue, the metatarsal exhibits two particular features that are possibly linked to the development of the vascularised endosteal bone. As previously described, the cancellous bone of the metatarsal reaches the outer cortex in particular region of the element, which is also characterized by the presence of a distinct excavation (Fig. 1a). The preserved primary bone tissue of this area is also distinctive in being composed of coarse bundles of mineralized fibres. These characteristics suggest a possible pathological origin of this tissue, and it is therefore likely that the vascularised endosteal tissue in the metatarsal is pathological.

The presence of vascularised endosteal bone tissue in the saltasaurine osteoderm poses an enigma. Indeed such tissue has been observed in osteoderms of fossil Archosauriformes. Scheyer *et al.*^17^ described pockets of convoluted well-vascularised secondary bone within an osteoderm of the aetosaur, *Calyptosuchus wellesi*, and Cerda *et al.*^19^ described endosteally formed bony trabeculae consisting of woven- and parallel-fibered bone within a cavity of an osteoderm of the doswelliid, *Tarjadia*. Several authors have proposed that the large cavities located within titanosaur osteoderms are suggestive of their role in calcium mobilization^20^,^33^,^38^. However, although crocodiles utilize calcium from their osteoderms they do not first deposit a special tissue (such as the high vacularised endosteal tissue of medullary bone), which is then mobilized. They appear to use the existing bone within the osteoderms, which result in the extensive remodelling of the osteoderms (as has been noted by several researchers).

Recently Prondvai and Stein^18^ described a “medullary-like” bone tissue in the mandibles of an azdarchid pterosaur (*Bakonydraco galaczi*). These researchers suggested the possibility that the tissue could be non-pathological since it occurs in four out of seven mandibles of the pterosaur, and they further suggested that it is not in response to reproduction since three of the specimens are fast growing immature individuals. However, given that outbreaks of disease are known to affect several individuals of wild populations^56^, it is possible that this tissue could reflect a pathological response in the four individuals. This tissue^18^ appears to resemble the vascularised endosteal bone tissue we describe in the saltasaurine titanosaur osteoderm and vertebra.

Our data suggests that only in the metatarsal can we deduce with independent evidence that the endosteal bone tissue is formed as a result of pathology. Vascularised endosteal bone tissue has been recorded and interpreted as being pathological in osteoderms of other Archosauriformes^17^,^19^. A similar tissue was described by Reid^15^ as a pathology in a pneumatic cavity of a sauropod vertebrae. Given that these previous studies have not provided any independent evidence for these being pathological, we cannot use them in support of a pathological origin for the observations made in our specimens. However, the finding of such endosteal bone tissue in our specimens, and possibly in the *Bakonydraco*^18^ suggests that such bone tissues appears to be fairly widespread within the phylogeny of Archosauriformes.

In conclusion, our study shows that the main criteria for the identification of medullary bone in fossil taxa i.e. location, origin, histology (including extent of mineralisation and birefringence patterns), cannot be employed because they also fit for different types of pathologies. For this reason, independent evidence is needed to assert either a pathological and/or reproductive origin for the occurrence of well-vascularised endosteal bone tissues. For example such evidence to support a pathological origin, could be corresponding periosteal reactive bone^14^,^16^, and/or lack of sexual maturity^21^, whereas independent evidence to support medullary bone could be morphometric studies^57^ or other evidence to support the identification of females^10^, or presence of eggs in the oviduct^2^. In the saltasaurine titanosaur samples studied here, direct independent evidence for a pathological cause of the well-vascularised endosteal bone tissue is only evident for the metatarsal. Interestingly in all the previous reports of vascularised endosteal tissue (Table 1), independent evidence besides the location, origin and histology has also only been provided in those specimens for which a pathological hypothesis is supported. To date, there is no independent evidence to support the presence of a tissue homologous to avian medullary bone in nonavian archosaurs.

## Additional Information

**How to cite this article**: Chinsamy, A. *et al.* Vascularised endosteal bone tissue in armoured sauropod dinosaurs. *Sci. Rep.* **6**, 24858; doi: 10.1038/srep24858 (2016).

## Supplementary Material

Supplementary Information

## Figures and Tables

**Table 1:** Occurrence of highly vascularized endosteal bone tissue in Archosauriformes.

Taxa	Element	Endosteal Reaction	Periosteal Reaction	Cause	Ref.
*Tarjadia*	osteoderm	X	–	Uncertain	19
*Calyptosuchus*	osteoderm	X	_	Uncertain	17
*Bakonydraco*	mandible	X	–	Non-pathological*	18
*Pterodaustro*	femur	X	_	Reproductive+	58
*Mussaurus*	femur	X	Uncertain	Pathological	16
*Allosaurus*	tibia	X	X	Reproductive^	12
*Allosaurus*	phalanx	X	X	Pathological	20
*T. rex*	tibia	X	_	Pathological	21
*T. rex*	femur and tibia	X	_	Reprodutive	9
Wealden sauropod	vertebrae	X	–	Pathology	15
*Tenontosaurus*	tibia and femur	X	–	Reproductive	12
*Dysalotosaurus*	tibia	X	_	Reproductive	11
Transylvanian dinosaur	femur	X	X	Pathology	14
*Stegosaurus*	tibia	X	–	Pathology	23
*Saltasaurus*	vertebrae	X	–	Uncertain	#
	osteoderm	X	–	Uncertain	#
	metatarsal	X	X	Pathological	#

^*^original authors suggest nonpathological, but current study proposes pathological.

^^^original authors suggest reproductive, but others (14) propose pathological.

^+^original authors proposed possibly reproductive but were uncertain.

^#^current study.
