# Supplementary material for: Vascularised endosteal bone tissue in armoured sauropod dinosaurs
Source: Sci Rep. 2016 Apr 26;6:24858. doi: 10.1038/srep24858 (PMC4845056; doi:10.1038/srep24858)
Supplement: Supplementary Information [file srep24858-s1.doc]

Vascularised endosteal bone tissue in armoured sauropod dinosaurs.

Anusuya Chinsamy^1^, Ignacio Cerda^2^ and Jaime Powell^3^

1 University of Cape Town, Department of Biological Sciences, Private Bag X3, Rhodes Gift, 7700 South Africa. Anusuya.chinsamy-turan@uct.ac.za

2 CONICET, Instituto de Investigación en Paleobiología y Geología, Universidad Nacional de Río Negro, Museo Carlos Ameghino, Belgrano 1700, Paraje Pichi Ruca (predio Marabunta) 8300, Cipolletti, Río Negro, Argentina, [nachocerda6@yahoo.com.ar](mailto:nachocerda6@yahoo.com.ar)

3 CONICET, Facultad de Ciencias Naturales Universidad Nacional de Tucumán, Miguel Lillo 205, (4000) Tucumán, Argentina. powell.jaime@gmail.com

SUPPLEMENTARY INFORMATION

**Supplementary Table S1: list of all elements sectioned and analyzed in this study.**

| **Taxa** | **Element** | **Collection number** | **Type of endosteal bone** |
| --- | --- | --- | --- |
| *Saltasaurus* | Humerus | PVL 4017-65 | Lamellar |
| *Saltasaurus* | Humerus | PVL 4017-62 | Lamellar |
| *Saltasaurus* | Humerus | PVL 4017-71 | Lamellar |
| *Saltasaurus* | Radius | PVL 4017-151 | Lamellar |
| *Saltasaurus* | Ulna | PVL 4017-75 | Lamellar |
| *Saltasaurus* | Ulna | PVL 4017-189 | Lamellar |
| *Saltasaurus* | Ulna | PVL 4017-219 | Lamellar |
| *Saltasaurus* | Scapula | PVL 4017-157 | Lamellar |
| *Saltasaurus* | Scapula | PVL 4017-153 | Lamellar |
| *Saltasaurus* | Sternal plate | PVL 4017-111 | Lamellar |
| *Saltasaurus* | Femur | PVL 4017-82 | Lamellar |
| *Saltasaurus* | Tibia | PVL 4017-220 | Lamellar |
| *Saltasaurus* | Tibia | PVL 4017-216 | Lamellar |
| *Saltasaurus* | Fibula | PVL 4017-91 | Lamellar |
| *Saltasaurus* | Metatarsal | PVL 4017-127 | Lamellar bone; vascularised woven fibered and parallel-fibered bone |
| *Saltasaurus* | Ilium | PVL 4017-221 | Lamellar |
| *Saltasaurus* | Ilium | PVL 4017-155 | Lamellar |
| *Saltasaurus* | Pubis | PVL 4017-103 | Lamellar |
| *Saltasaurus* | Pubis | PVL 4017-222 | Lamellar |
| *Saltasaurus* | Ischium | PVL 4017-223 | Lamellar |
| *Saltasaurus* | Ischium | PVL 4017-158 | Lamellar |
| *Saltasaurus* | Ischium | PVL 4017-224 | Lamellar |
| *Saltasaurus* | Dorsal rib | PVL 4017-148 | Lamellar |
| *Saltasaurus* | Dorsal rib | PVL 4017-146 | Lamellar |
| *Saltasaurus* | Dorsal rib | PVL 4017-144 | Lamellar |
| *Saltasaurus* | Dorsal rib | PVL 4017-171 | Lamellar |
| *Saltasaurus* | Dorsal rib | PVL 4017-143 | Lamellar |
| *Saltasaurus* | Dorsal rib | PVL 4017-225 | Lamellar |
| *Saltasaurus* | Dorsal rib | PVL 4017-226 | Lamellar |
| *Saltasaurus* | Posterior caudal vertebra | PVL 4017-140 | Lamellar bone; Vascularised woven fibered and paralll fibered bone |
| *Saltasaurus* | Posterior caudal vertebra | PVL 4017-207 | Lamellar |
| *Saltasaurus* | Mid caudal vertebra | PVL 4017-56 | Lamellar |
| *Saltasaurus* | Cervical vertebra | PVL 4017-217 | Lamellar |
| *Saltasaurus* | Mid caudal vertebra | PVL 4017-192 | Lamellar |
| *Saltasaurus* | Osteoderm | PVL 4017-113 | Lamellar bone; Vascularised woven fibered and parallel fibered bone |
| Lithostrotia indet. | Osteoderm | MCS-Pv 181 | Lamellar |
| Lithostrotia indet. | Osteoderm | MCS-Pv 182 | Lamellar |
| Lithostrotia indet. | Osteoderm | MCS-Pv 62 | Lamellar |
| Lithostrotia indet. | Osteoderm | MPCA-Pv 67 | Lamellar |
| Lithostrotia indet. | Osteoderm | MPCA-Pv sn | Lamellar |
| *Neuquensaurus* | Humerus | MLP CS 1009 | Lamellar |
| *Neuquensaurus* | Ulna | MLP CS 1094 | Lamellar |
| *Neuquensaurus* | Scapula | MLP CS 1129 | Lamellar |
| *Neuquensaurus* | Femur | MCS-Pv 5/28 | Lamellar |
| *Neuquensaurus* | Tibia | MPCA-Pv CS 002 | Lamellar |
| *Neuquensaurus* | Tibia | MLP CS 1093 | Lamellar |
| *Neuquensaurus* | Tibia | MLP CS 1303 | Lamellar |
| *Neuquensaurus* | Metatarsal | MCS-Pv 174/11 | Lamellar |
| *Neuquensaurus* | Ilium | MLP-CS 1056 | Lamellar |
| *Neuquensaurus* | Ischium | MPCA-CS 001 | Lamellar |
| *Neuquensaurus* | Dorsal rib | MCS-Pv 5/36 | Lamellar |
| *Neuquensaurus* | Dorsal rib | MCS-Pv 5/37 | Lamellar |
| *Neuquensaurus* | Dorsal rib | MCS-Pv 174/12 | Lamellar |
| *Neuquensaurus* | Caudal vertebra | MPCA-Pv sn | Lamellar |
| *Neuquensaurus* | Caudal vertebra | MCS-Pv 180 | Lamellar |
| *Neuquensaurus* | Caudal vertebra | MPCA-Pv Cs 003 | Lamellar |
| *Neuquensaurus* | Caudal vertebra | MPCA-Pv Cs 006 | Lamellar |
| *Neuquensaurus* | Caudal vertebra | MPCA-Pv Cs 004 | Lamellar |
| *Neuquensaurus* | Caudal vertebra | MCS-Pv 174/13 | Lamellar |
| *Neuquensaurus* | Caudal vertebra | MPVA-Pv Cs 005 | Lamellar |
| *Neuquensaurus* | Chevron | MCS-Pv 5/32 | Lamellar |
| *Neuquensaurus* | Chevron | MLP CS 1245 | Lamellar |
| *Andesaurus* | Femur | MUCPv 132 | Lamellar |
| *Andesaurus* | Dorsal rib | MUCPv 132 | Lamellar |
| *Andesaurus* | Dorsal rib | MUCPv 132 | Lamellar |
| *Argentinosaurus* | Dorsal rib | PVPH-1 | Lamellar |
| *Laplatasaurus* | Fibula | MUCPv 239 | Lamellar |
| *Laplatasaurus* | Metacarpal | MUCPv 239 | Lamellar |
| *Laplatasaurus* | Ilium | MUCPv 239 | Lamellar |
| *Aeolosaurus* sp. | Femur | MPCA-Pv 27100 | Lamellar |
| *Aeolosaurus* sp. | Sternal plate | MPCA-Pv 27100 | Lamellar |
| *Aeolosaurus* sp. | Dorsal rib | MPCA-Pv 27100 | Lamellar |
| *Aeolosaurus* sp. | Ulna | MPCA-Pv sn | Lamellar |
| *Aeolosaurus* sp. | Mid caudal vertebra | MPCA-Pv sn | Lamellar |

Institutional Abreviations: MCF-PVPH, Vertebrate Paleontology of Museo Carmen Funes, Plaza Huincul, Argentina; MCS-Pv, Vertebrate Paleontology of Museo de Cinco Saltos, Río Negro, Argentina; MLP-CS, Museo de La Plata, Cinco Saltos collection, La Plata, Argentina; MPCA-Pv, Vertebrate Paleontology of Museo Provincial ‘Carlos Ameghino,’ Cipolletti, Argentina; MUCPv, Vertebrate Paleontology of Museo de la Universidad Nacional del Comahue, Neuquén, Argentina; PVL, Vertebrate Paleontology of Fundacion Miguel Lillo, Universidad Nacional de Tucumán, San Miguel de Tucumán, Argentina.


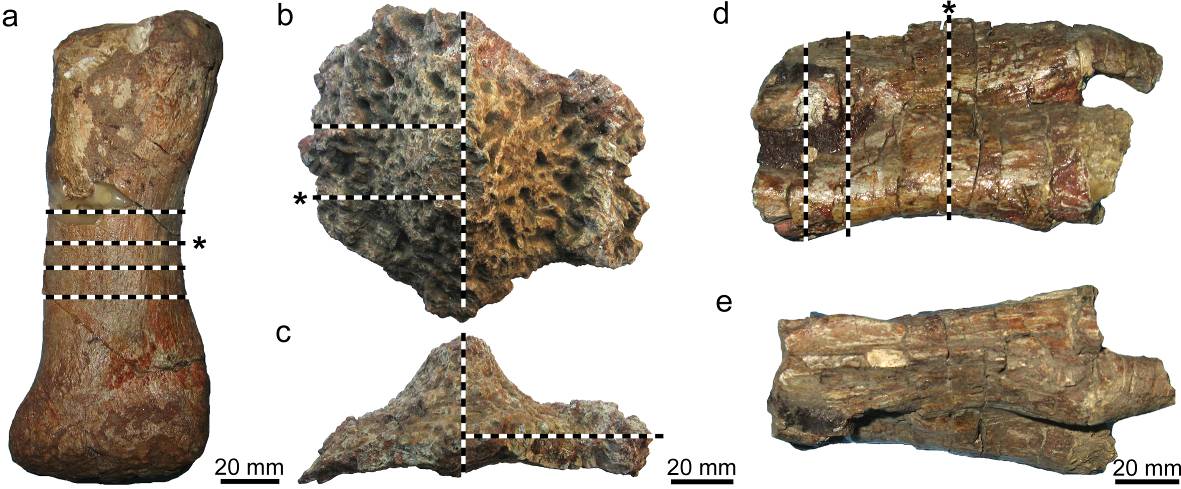


**Supplementary Figure 1**: Metatarsal PVL 4017-127 (**a**), osteoderm PVL 4017-113 (**b**, dorsal view; **c**, lateral view) and posterior caudal vertebra PVL 4017-140 (**d**, lateral view; **e**, dorsal view) in which unusual endosteal bone tissue was recognized. Dashed lines show the location and orientation of the thin sections. Asterisk (*) indicates the thin sections in Figures 1a, 2a and 3a.


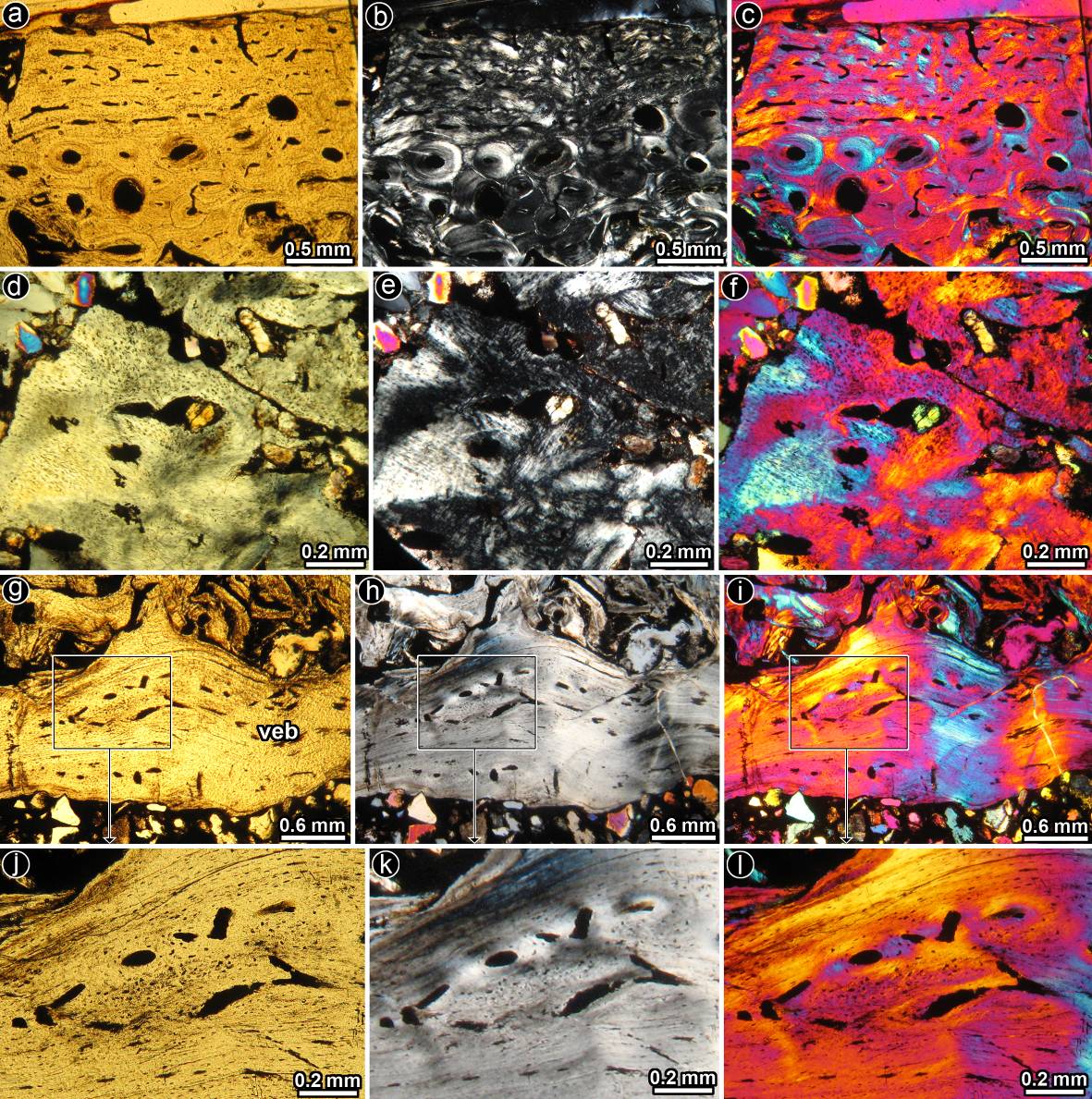


**Supplementary Figure 2**: Metatarsal PVL 4017-127 bone histology. **a,bc**: general view of the primary bone tissue. **d,e,f**: detailed view of the highly fibrous bone tissue at the outer cortex. **g,h,i**: general view of the vascularised endosteal bone (veb) tissue at the medullary region. **j,k,l**: detailed view of the vascularised bone tissue at the medullary region. **a,d,g,j:** normal transmitted light, **b,e,h,k:** polarized light, **c,f,i,l:** polarized light with lambda compensator.


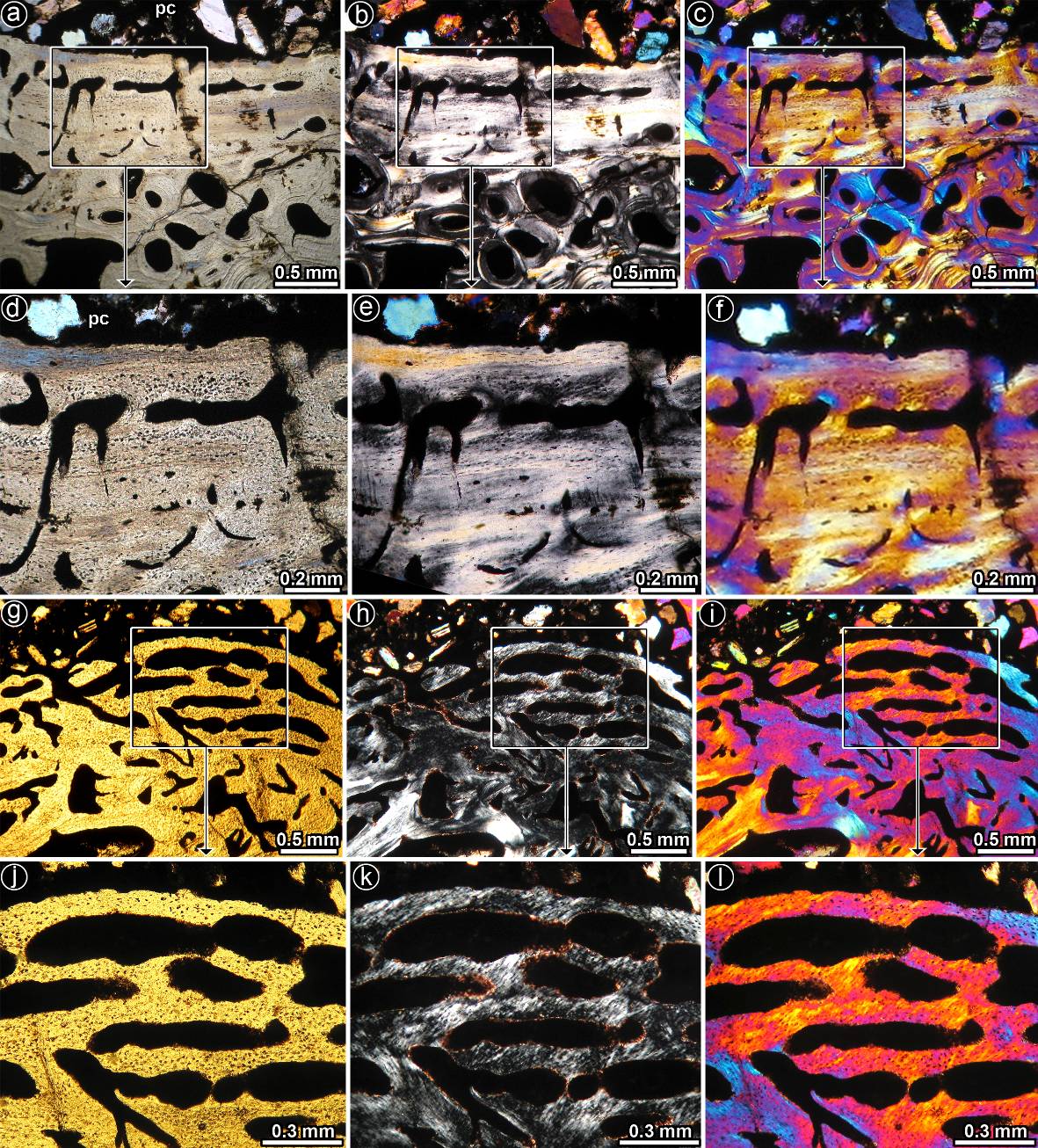


**Supplementary Figure 3**: bone histology of posterior caudal vertebra PVL 4017-140 (**a-f**) and osteoderm PVL 4017-113 (**g-l**). **a,b,c**: general view of the vascularised endosteal bone tissue around the pneumatic cavity. **d,e,f**: detailed view of the vascularised endosteal bone tissue around the pneumatic cavity. **g,h,i**: general view of the vascularised endosteal bone tissue around an internal cavity. **j,k,l**: detailed view of the vascularised endosteal bone tissue around an internal cavity. **a,d,g,j:** are under normal transmitted light, **b,e,h,k:** cross polarized light, **c,f,i,l:** polarized light with lambda compensator.


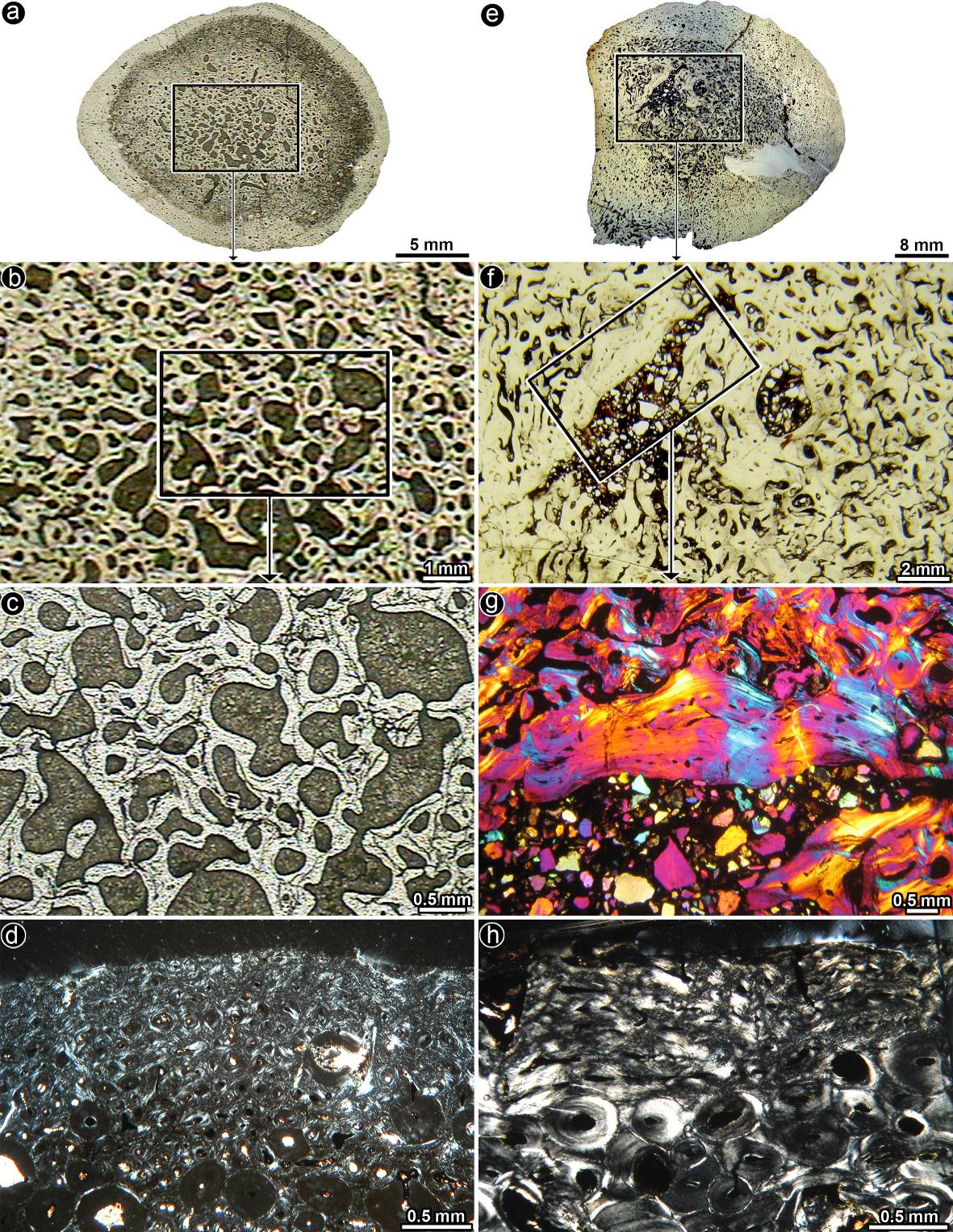


**Supplementary Figure 4**: Comparative histology between a titanosaur sauropod (possibly *Neuquensaurus*) MCS Pv 174/11 (**a-d**) and metatarsal PVL 4017-127 (**e-h**). **a,d**: complete transverse section at level of the midshaft. **b,f**: general view of the medullary region. **c,g**: detailed view of the medullary region. Note the presence of vascularised endosteal bone in g. **d,h**: general view of the primary bone tissue at the outer cortex. **a,b,c,f:** are under normal transmitted light, **d,h:** cross polarized light, **g:** polarized light with lambda compensator.


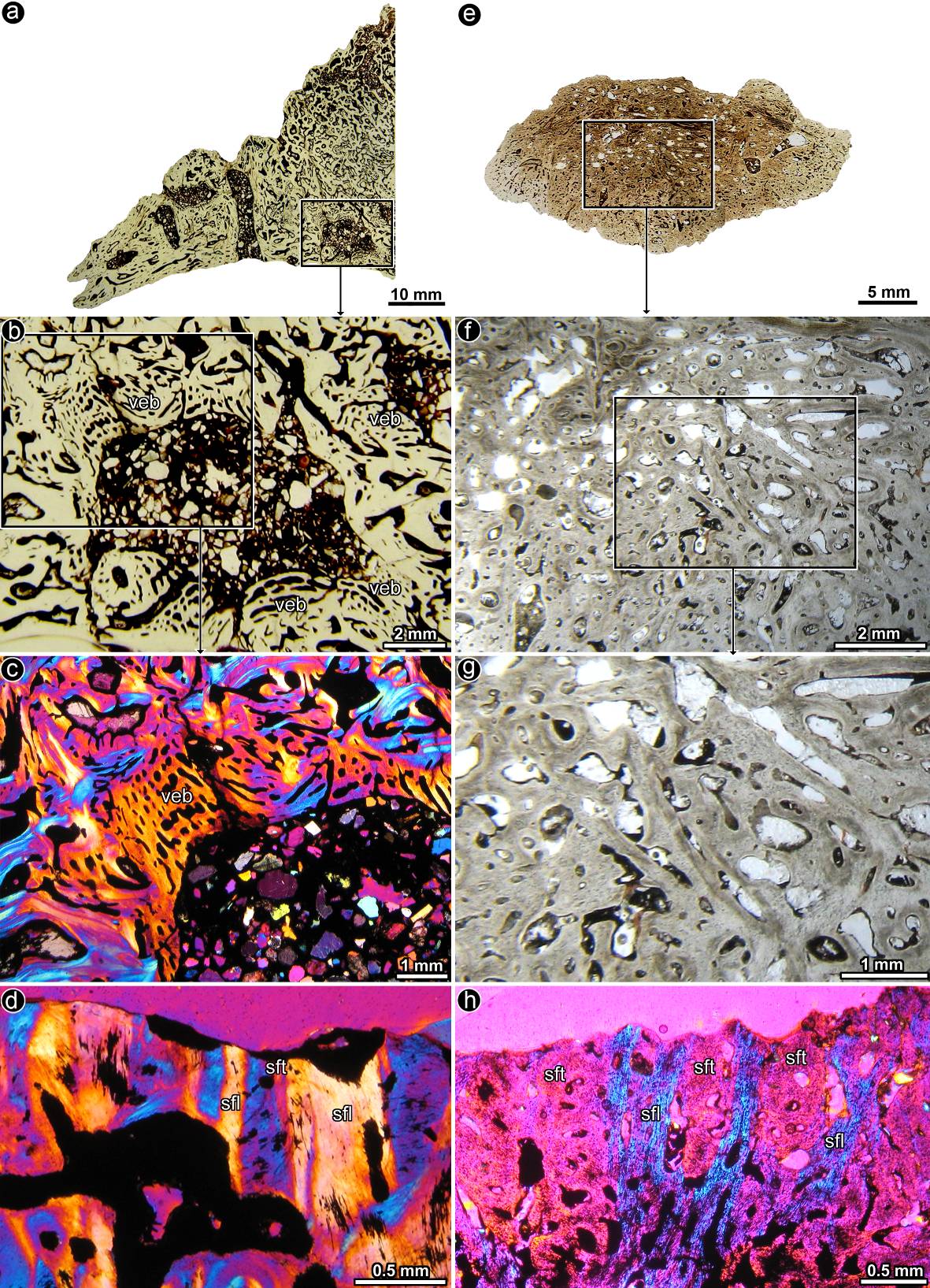


**Supplementary Figure 5**: Comparative histology of the osteoderm PVL 4017-113 (**a-d**) and an undetermined titanosaur sauropod MCS Pv 181 (**e-h**). **a,e**: half (**a**) and complete (**d**) cross sections. **b,f**: general view of the internal structure. Note the abundance of vascularised endosteal bone (veb) in PVL 4017-113. **c,g**: detailed view of the internal region of the osteoderms. **d,h**: detailed view of the preserved structural fiber bundles (primary bone formed by metaplasia) in the external cortex. Structural fibers have been both transversally (sft) and longitudinally (sfl) sectioned. **a,b,e,f,g:** are under normal transmitted light; **c,d,h:** are under polarized light with lambda compensator.


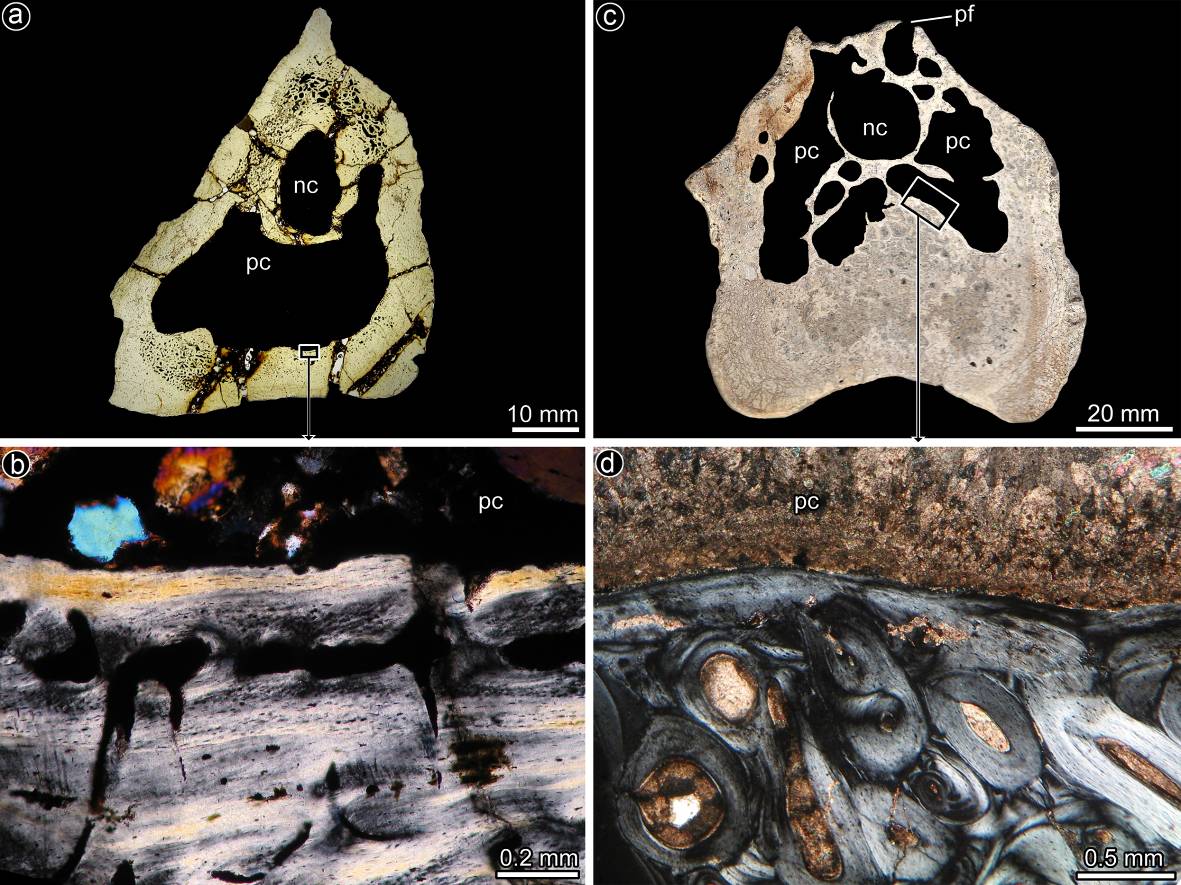


**Supplementary Figure 6**: Comparative histology between caudal vertebra PVL 4017-140 (**a-b**) and caudal vertebra MCS Pv 180 (**c,d**), which is assigned to *Neuquensaurus australis*. a,b: complete cross section of the vertebrae. Sediment in both neural canal (nc) and pneumatic cavities (pc) has been digitally erased. A pneumatic foramen (pf) is denoted in the neural arch of *Neuquensaurus*. **c,d**: detailed view of the bone tissue around pneumatic cavities, **a,c:** normal light, **b,d:** cross polarized light.
